# Exploring venlafaxine pharmacokinetic variability with a phenotyping approach, a multicentric french-swiss study (MARVEL study)

**DOI:** 10.1186/s40360-017-0173-2

**Published:** 2017-11-07

**Authors:** Célia Lloret-Linares, Youssef Daali, Sylvie Chevret, Isabelle Nieto, Fanny Molière, Philippe Courtet, Florence Galtier, Raphaëlle-Marie Richieri, Sophie Morange, Pierre-Michel Llorca, Wissam El-Hage, Thomas Desmidt, Frédéric Haesebaert, Philippe Vignaud, Jerôme Holtzmann, Jean-Luc Cracowski, Marion Leboyer, Antoine Yrondi, Fabienne Calvas, Liova Yon, Philippe Le Corvoisier, Olivier Doumy, Kyle Heron, Damien Montange, Siamak Davani, Julien Déglon, Marie Besson, Jules Desmeules, Emmanuel Haffen, Frank Bellivier

**Affiliations:** 1 Inserm, U1144, F-75006 Paris, France; 2 Université Paris Diderot, UMR-S 1144, F-75013 Paris, France; 3 Department of Internal Medicine, Assistance Publique-Hôpitaux de Paris, Hôpital Lariboisière, Therapeutic Research Unit, F-75010 Paris, France; 4 Division of Clinical Pharmacology and Toxicology, Geneva University Hospitals, Geneva, Switzerland; 5 Service de Biostatistiques et Information Médicale, Hôpital Saint-Louis, AP-HP, ECSTRA Team, Inserm UMR-1153, Université Paris Diderot, 1 rue Claude Vellefaux, 75010 Paris, France; 6 Department of Psychiatry and Addiction Medicine, Assistance Publique-Hôpitaux de Paris, Hôpital F. Widal, F-75010 Paris, France; 7 CHRU Montpellier, Mont-Saint-Éloi, France; 8 Pôle psychiatrie, addictologie, pédopsychiatrie, Assistance Publique des hôpitaux de Marseille, Marseille, France; 9 APHM, Aix Marseille Univ, Institut Paoli-Calmettes, INSERM, CIC Hôpital Conception, Marseille, France; 10 Service Psychiatrie et Addictologie de l’Adulte CMP B, Centre Hospitalier Universitaire, Rue Montalembert, Clermont-Ferrand, France; 11 Inserm U930, Université François Rabelais de Tours, Tours, France; 12 Inserm CIC 1415, Tours, France; 13 Clinique Psychiatrique Universitaire, CHRU de Tours, Tours, France; 14 PsyR2 Team, U 1028, INSERM and UMR 5292, CNRS, Center for Neuroscience Research of Lyon (CRNL), CH Le Vinatier, Lyon-1 University, Bron, France; 15 Centre Interdisciplinaire de Recherche en Réadaptation et en Intégration Sociale (CIRRIS), Centre de Recherche de l’Institut Universitaire en Santé Mentale (CRIUSM), Université Laval, QC, Québec Canada; 16 Service Hospitalo-Universitaire de Psychiatrie. CHU Grenoble-Alpes, La Tronche, France; 17 Unité de Pharmacologie Clinique, Centre d’Investigation Clinique de Grenoble, INSERM CIC1406, CHU de Grenoble, Grenoble, France; 18 Service de psychiatrie et psychologie médicale CHU Toulouse-Purpan, Toulouse, France; 19 Toulouse NeuroImaging Center, ToNIC, University of Toulouse, Inserm, UPS, Toulouse, France; 20 AP-HP, pole de psychiatrie des HU Henri Mondor, Equipe psychiatrie translationnelle, Créteil, France; 21 Inserm U955 and foundation FondaMental, Créteil, France; 22 Inserm CIC 1436, CHU Toulouse, Université Toulouse III Paul Sabatier, Toulouse, France; 23 Inserm, Clinical Investigation Center 1430 and Henri Mondor University Hospital, AP-HP, Créteil, France; 24 Centre Expert Dépression Résistante, Centre Référence Pathologies Anxieuses et Dépression (CERPAD), Centre Hospitalier Charles Perrens, Bordeaux, France; 25 Department of Experimental Psychology, University of Bristol, UK and Somerset Partnership NHS Foundation Trust, Bristol, UK; 26 Department of Pharmacology, CHRU Besançon, Univ. Bourgogne-Franche-Comté, EA3920 Besançon, France; 27 Unit of Toxicology, CURML, University Hospitals of Lausanne, Lausanne, Switzerland; 28 Unit of Toxicology, CURML, University Hospitals of Geneva, Geneva, Switzerland; 29 Department of Clinical Psychiatry, University Hospital of Besançon, Besançon, France

## Abstract

**Background:**

It is well known that the standard doses of a given drug may not have equivalent effects in all patients. To date, the management of depression remains mainly empirical and often poorly evaluated. The development of a personalized medicine in psychiatry may reduce treatment failure, intolerance or resistance, and hence the burden and costs of mood depressive disorders.

The Geneva Cocktail Phenotypic approach presents several advantages including the “in vivo” measure of different cytochromes and transporter P-gp activities, their simultaneous determination in a single test, avoiding the influence of variability over time on phenotyping results, the administration of low dose substrates, a limited sampling strategy with an analytical method developed on DBS analysis.

The goal of this project is to explore the relationship between the activity of drug-metabolizing enzymes (DME), assessed by a phenotypic approach, and the concentrations of Venlafaxine (VLX) + O-demethyl-venlafaxine (ODV), the efficacy and tolerance of VLX.

**Methods/design:**

This study is a multicentre prospective non-randomized open trial. Eligible patients present a major depressive episode, MADRS over or equal to 20, treatment with VLX regardless of the dose during at least 4 weeks. The Phenotype Visit includes VLX and ODV concentration measurement.

Following the oral absorption of low doses of omeprazole, midazolam, dextromethorphan, and fexofenadine, drug metabolizing enzymes activity is assessed by specific metabolite/probe concentration ratios from a sample taken 2 h after cocktail administration for CYP2C19, CYP3A4, CYP2D6; and by the determination of the limited area under the curve from the capillary blood samples taken 2–3 and 6 h after cocktail administration for CYP2C19 and P-gp.

Two follow-up visits will take place between 25 and 40 days and 50–70 days after inclusion. They include assessment of efficacy, tolerance and observance.

Eleven french centres are involved in recruitment, expected to be completed within approximately 2 years with 205 patients. Metabolic ratios are determined in Geneva, Switzerland.

**Discussion:**

By showing an association between drug metabolism and VLX concentrations, efficacy and tolerance, there is a hope that testing drug metabolism pathways with a phenotypical approach would help physicians in selecting and dosing antidepressants. The MARVEL study will provide an important contribution to increasing the knowledge of VLX variability and in optimizing the use of methods of personalized therapy in psychiatric settings.

**Trial registration:**

ClinicalTrials.gov NCT02590185 (10/27/2015). This study is currently recruiting participants.

## Background

### Challenge in depression management

A recent epidemiological study has shown major depressive disorder (MDD) to be associated with a significant morbidity burden; it has the second largest proportion of individuals living with disability worldwide and in this respect is second only to low back pain [1]. Mood disorders are the most prominent and the most expensive brain disorders in Europe [2]; the total annual cost per disorder was €113.4 billion in 2010, slightly higher than dementia, but tenfold the cost of epilepsy or Parkinson’s disease and two-fold the cost of stroke [3]. Direct cost constituting the majority of the total cost with the remainder being attributable to indirect costs associated with patients’ production losses.

All costs are increased due to unpredictable response to antidepressant therapy [4]; despite the availability of an increasing number of pharmacological treatments for MDD, only 25 to 35% of the patients recover fully from a depressive episode after first line treatment, necessitating either a trial of a second antidepressant or an augmentation strategy. In addition, many patients do not recover to a durable, long-term functional remission [5, 6]. Patients with resistance to treatment are twice as likely to be hospitalized, have more outpatient visits, use more psychotropic medications, and have 19 times the depression-related costs compared to patients with depression that responds to treatment [7].

It is well known that the standard doses of a given drug may not have equivalent effects in all patients. To date, the management of depression remains mainly empirical and often poorly evaluated. Thus the costs associated with MDD might be mitigated by the individualization of its treatment; the development of a personalized medicine in psychiatry may reduce treatment failure, intolerance or resistance, and hence the burden and costs of MDD.

### Emergence of pharmacokinetic biomarkers of antidepressant efficacy

Progress towards individualization requires an understanding of the origins of response variability and the development of strategies to manage it. Factors that cause variability in antidepressant response are complex; they include modifiable and non-modifiable, pharmacokinetic (PK) and pharmacodynamic (PD) factors [8]. PK factors are emerging as attractive predictive markers of drug response, particularly as no suitable markers related to antidepressant mechanisms of action have so far been identified. Studies of different classes of antidepressants in both clinical trials and clinical settings have shown a relationship between drug concentrations, the magnitude and the duration of pharmacologic effects; thus an understanding of an individual PK profile may allow antidepressant response variability to be accounted for in the choice of therapeutic agent.

The GeneSight pharmacogenomic test and interpretive report has been designed to predict antidepressant responses based on DNA variations in cytochrome P450 genes (CYP2D6, CYP2C19, CYP2C9 and CYP1A2), the serotonin transporter gene (SLC6A4) and the serotonin 2A receptor gene (5HTR2A) [9]. This algorithm is based on the genotyping of both copies of five pharmacokinetic and pharmacodynamic genes selected for their relevance to clinical response to antidepressants and antipsychotics.

Three prospective clinical studies confirmed the benefit of such investigations for the management treatment-resistant depression. The odds of clinical response were increased 2.3-fold among all GeneSight-guided treatments compared to all treated as usual subjects (*p* = 0.004), the guided group had a 53% greater improvement in depressive symptoms (*p* = 0.0002), a 1.7-fold relative improvement in response (*p* = 0.01), and a number needed to treat for one clinical response above that seen in the treated as usual group of 6.07 [10].

In a retrospective study, Winner et al. evaluated eight direct or indirect health care utilization measures for 96 patients with a diagnosis of depressive or anxiety disorder [11]. The eight measures were evaluated in relation to the pharmacogenomics test and reporting system. Subjects whose medication regimen included a medication identified by the test as most problematic (medication status of ‘use with caution and frequent monitoring’), had 69% more total health care visits, 67% more general medical visits, greater than three-fold more medical absence days, and greater than four-fold more disability claims than subjects taking drugs categorized as ‘use as directed’ or ‘use with caution’. The test can identify past inappropriate medication selection, which led to increased healthcare utilization and cost. Moreover, pharmacogenomics test in patients who had switched or added a new psychiatric medication after having failed monotherapy provides significant ‘real world’ cost savings, while simultaneously improving adherence [12].

### Clinical investigation of drug metabolism

Drug metabolism may be affected to varying degrees by physiological and pathological factors and by drug-drug interactions involving metabolizing enzymes as well as by genetic polymorphism.

#### Genotyping

The activity of the enzymes and transporters involved in drug PK parameters may be influenced by their genetic variation. For a subset of alleles in vivo and in vitro studies have elucidated enzyme activities that are listed as increased, normal, decreased, absent or unknown. This list is available in *‘The Human Cytochrome P450 (CYP) Allele Nomenclature Database’* (http://www.PharmVar.org), which catalogues genetic variability in CYP enzymes. This information can be used, along with the number of functional alleles and the presence of gene duplications, to predict the metabolic phenotypes.

Poor or slow metabolizers (PMs) have deficient metabolizing ability compared with persons with normal activity. PMs relative to more rapid metabolizers are more likely to suffer from adverse drug effects when taking normal doses of drugs that are active per se and are metabolized mainly via these pathways. Conversely, an increased amount of active metabolites in ultrarapid metabolizers (UMs) may induce side effects resulting in reduced tolerability. Furthermore in UMs increased metabolism of active drug to inactive metabolite is likely to result in a reduced efficacy relative to the slower metabolic phenotypes. Between these two extreme profiles, intermediate (IMs) and extensive (EMs) profiles have also been described. The distribution of phenotypes differs according to ethnic origin [13]. Moreover, the number of phenotypes depends on the CYP; for certain CYP’s and for the P-glycoprotein (P-gp), three phenotypes of activity are described (reduced, normal, induced).

#### Phenotyping

Approaches based on gene polymorphism identification may not provide an accurate estimate because of poor genotype/phenotype correlation for some genes in certain clinical situations [13–19]. The phenotypic approach can effectively assess Drug Metabolizing Enzyme (DME) activity independently of a specific treatment, even before starting it [9, 20, 21]. Phenotyping consists of the administration of probe substrates metabolised by a specific CYP or transported by P-gp, followed by the determination of a metabolic ratio (MR) or the evaluation of the plasma or urine probes’ concentrations. The major strength of this approach is the direct measure of CYP activity, including genetic, physiological and environmental factors. Classification by phenotype is based on drug concentration or blood or urinary metabolic ratio [22, 23]. Phenotyping tests can be either individual or simultaneous; individual phenotyping involves administration of one CYP specific probe, while simultaneous phenotyping involves concomitant administration of multiple specific probes (probe cocktail) and allows the detection of the activity of multiple enzymes simultaneously.

### Venlafaxine

Venlafaxine (VLX) is a serotonin–norepinephrine reuptake inhibitor marketed for the treatment of depression disorders. It provides a reasonable second-step choice for patients with depression and is used extensively in psychiatric practice [24, 25]. Regarding differences in efficacy and tolerability between “newer” antidepressants, Cipriani et al. found in a meta-analysis of 117 randomized clinical trials with 25,928 patients that mirtazapine, escitalopram, VLX, and sertraline were significantly more efficacious than duloxetine, fluoxetine, fluvoxamine, paroxetine, and reboxetine [25].

#### Pharmacology

VLX is primarily metabolised into the active metabolite O-desmethyl-VLX (ODV), with serotonin and noradrenaline reuptake inhibition properties. The mean plasma half-lives (± SD) of VLX and ODV are 5(±2) hours and 11(±2) hours, respectively. Steady-state concentrations of VLX and ODV are attained within 3 days of oral multiple-dose therapy. VLX and ODV exhibit linear kinetics over the dose range of 75 mg to 450 mg/day [26]. Absolute bioavailability is 40% to 45% due to pre-systemic metabolism. VLX and ODV are minimally bound at therapeutic concentrations to human plasma proteins (25–30%, approximately). The volume of distribution for VLX at steady-state is 4.4 ± 1.6 L/kg following intravenous administration.

#### The therapeutic range of VEN + ODV in blood is between 125 and 400 μg/l [27].

VLX is highly metabolized in humans, with urinary excretion of the unchanged compound being between 1 and 10% of an administered dose [28]. Cytochrome P450 2D6 (CYP2D6) is the major enzyme involved in ODV formation, which is excreted unchanged and as its glucuronide. Despite the major role of CYP2D6, ODV concentrations are detectable in CYP2D6 PMs and CYP2C19 may also be involved in the formation of ODV to a minor extent [29, 30]. N-Demethylation of VLX to the inactive metabolite N-desmethyl-VLX (NDV) by CYP3A4 and CYP2C19 is generally a minor metabolic pathway [29, 31]. Patients with the CYP2D6 PM phenotype show a higher level of NDV compared with CYP2D6 EM patients, implicating an increase in flux through this route when ODV production is reduced [32, 33].

**Fig. 1:**
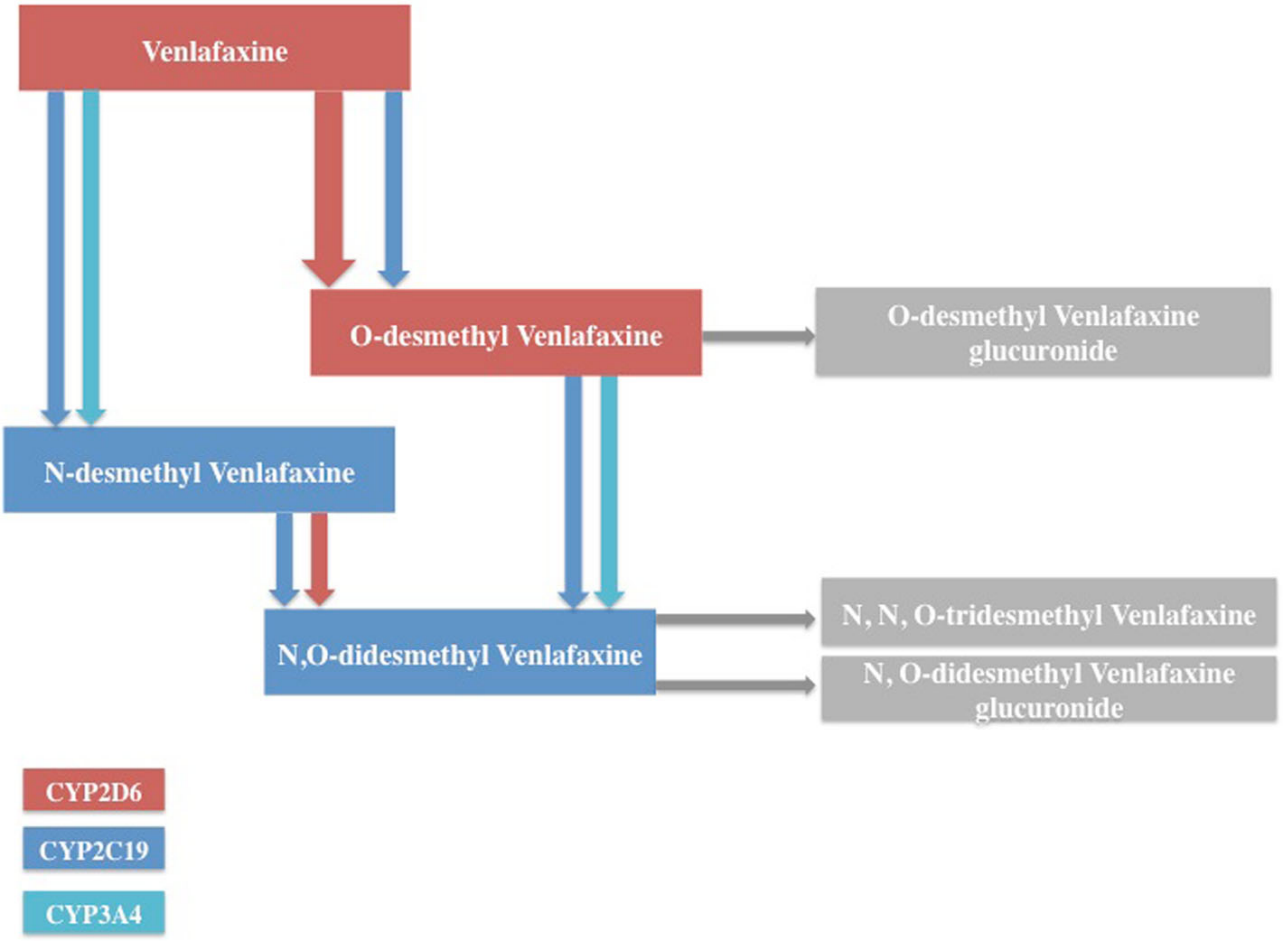
Venlafaxine metabolism

ODV and NDV are further metabolized by CYP2C19, CYP2D6, and/or CYP3A4 into N,O-didesmethyl-VLX, a minor metabolite with no known pharmacological effect, which is itself metabolized into N,N,O-tridesmethyl-VLX or excreted as its glucuronide (Fig. 1.).

#### CYP variability and VLX

The effect of CYP2C19 in VLX metabolism and efficacy has not been extensively studied to date. As both PM and UM variations of CYP2C19 are present in most populations, it is reasonable to expect that CYP2C19 variability may have an impact on VLX metabolism, particularly in patients carrying CYP2D6 PM and IM phenotypes. In addition, as VLX is a weak inhibitor of CYP2D6 and CYP3A4, CYP2C19 pathways are though to be significantly involved in ODV metabolism [34].

Some studies have indicated that polymorphisms in both CYP2D6 and CYP2C19 influenced VLX metabolism [8]. Moreover the combined missing CYP2D6 and CYP2C19 activity has been involved in the occurrence of a fatal drug poisoning case in a patient receiving VLX [35]. McAlpine et al. showed a significant positive effect of both CYP2D6 and CYP2C19 genotype scores on ODV/VLX ratio (CYP2D6: *r* = 0.44,*p* = 0.001; CYP2C19: *r* = 0.26, *p* = 0.009), consistent with the hypothesis that both enzymes are involved in VLX metabolism [36]. The highest ODV/VLX ratios were related to highest CYP2D6 activity. However, they also demonstrate that CYP2D6 and CYP2C19 allelic variants are independent predictors of lower total concentration (CYP2D6: *P* = 0.021, CYP2C19: *P* = 0.001).

But to date, no study has investigated the effect of variations in both CYP2D6 CYP2C19 activity on VLX efficacy and tolerance.

### Research objectives

The goal of this project is to explore the relationship between the activity of drug-metabolizing enzymes (DME) and transporters, assessed by a phenotypic approach, and the concentrations of VLX + ODV, the efficacy and tolerance of VLX.

### Primary objective

To study the correlations between the concentrations of VLX + ODV and drug metabolism variability as assessed by a phenotypic approach.

### Secondary objectives

i. To compare between responders and non-responders, as well as between patients with or without side effects:
  - The CYP2C19 activity and the prevalence of each profile of metabolism.
  - The CYP2D6 activity and the prevalence of each profile of metabolism.
  - The CYP3A4 activity and the prevalence of each profile of metabolism.
  - The P-gp activity and the prevalence of each profile of transport.

ii. To study the correlation between VLX + ODV concentration/dose and VLX + ODV concentration and antidepressant efficacy and tolerance.
iii. To study the correlation between the ratio ODV/VLX and CYP2D6 activity.
iv. To study the correlation between the concentration at 2 h and the AUC (2,3,6 h) of the metabolic ratio hydroxyomeprazole/omeprazole.
v. To conduct exploratory association analyses between blood biomarkers (candidate mRNA and miRNA) and the tolerance and efficacy of VLX.
vi. To analyse the role of genetic variations of DNA in the determination of CYP2C19 and 2D6 phenotypes, in patients with PM profile.

## Methods

This study is a multicentre prospective non-randomized open trial.

### Patients

In this study, male and female patients, aged from 18 to 80 years, with a major depressive episode meeting DSM-V criteria, will be eligible for participation.

Inclusion criteria include: 1) MADRS over or equal to 20 at selection visit, 2) treatment with VLX regardless of the dose during at least 4 weeks, 3) Decision of the treating psychiatrist to increase the dose of VLX at the selection visit, 5) Understanding of the French language and able to give written informed consent, 6) Informed consent to participation in the study signed, 7) Individuals covered by social security regimen.

Exclusion criteria include: 1) Patients treated by more than one antidepressant other than mirtazapine or mianserine, 2) Patients currently treated with one of the constituents of the substrate cocktail and/or by esomeprazole, 3) Sensitivity or contra-indications to any of the substrate drugs used, 4) Current pregnancy or intention to become pregnant, or breastfeeding, 5) Diagnosis of Bipolar disorder or schizophrenia.

### Recruitment and design

**Fig. 2:**
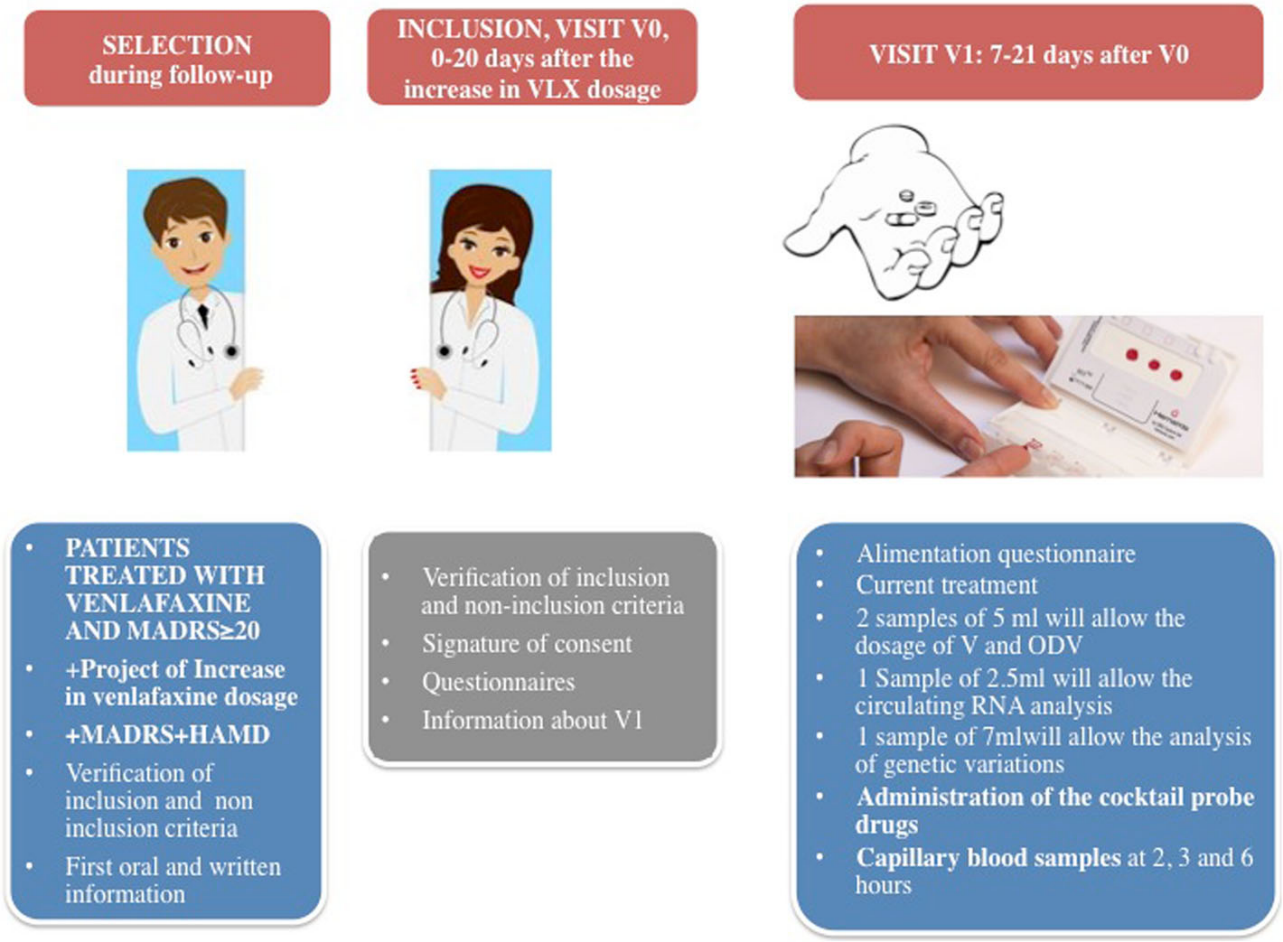
Summary of MARVEL design. V (Venlafaxine), ODV (O-demethyl-Venlafaxine). MADRS (Montgomery and Asberg Depression Rating Scale); HAM-D (Hamilton Rating Scale for Depression). V0 (Inclusion Visit), V1 (Phenotypic visit)

All 11 recruitment sites are coordinated by the FondaMental foundation (www.fondation.fondamental.org) and belong to the Network of expert centres for Resistant Depression. Comprehensive assessment is offered to patients with resistant depression and data are entered into dedicated web-based application (e-DR). Patients are referred to the expert centre by a general practitioner or a psychiatrist. Psychiatrists recruit patients in hospital or ambulatory setting. Two rating scales for depression are performed during the selection visit; the Montgomery and Asberg Depression Rating Scale (MADRS) and the Hamilton Rating Scale for Depression (HAM-D). Dependent upon psychiatric evaluation and in accordance with usual practice, the psychiatrist (and investigator) propose an increase in dosage if this is indicated. In this context, the investigator gives oral and written information about the study. Subsequent visits are planned as per Fig. 2.

#### Inclusion visit (V0)

The visit takes place **0–20 days** after the increase in VLX dosage (selection visit). The investigator obtains the written informed consent and several questionnaires are completed (Table 1).

**Table 1:** Study schedule. MADRS and HAM-D at V0 are part of usual care of the included patients

Criteria	Selection: Psychiatry consultation or hospitalization	Visit V0: Psychiatry 0–20 days after decision of drug dosage increase	Visit V1: With a nurse Period: 7–21 days after V0	Visit V2: With a Psychologist or a pratictionner: 25–40 days (4 weeks) after V0	Visit V3: With a Psychologist or a pratictionner: 50–70 days (8 weeks) after V0
Oral and written Information about the protocol	MARVEL	MARVEL			
Verification of inclusion and non inclusion criteria, Signature of informed consent	MARVEL	MARVEL			
MADRS and HAM-D		Usual care		MARVEL	MARVEL
Screen for tobacco use		MARVEL			
Renal and liver function		MARVEL			
Characteristics of the mood disorder		MARVEL			
Anxiety scale Tyrer		MARVEL			
Criteria for rating medication trials for antidepressant failure		MARVEL			
QIDS-score		MARVEL		MARVEL	MARVEL
MARS score		MARVEL		MARVEL	MARVEL
PRISE-M score		MARVEL		MARVEL	MARVEL
FIBSER score		MARVEL		MARVEL	MARVEL
Urinary pregnancy test			MARVEL		
Current treatment		MARVEL	MARVEL	MARVEL	MARVEL
Recent alimentation			MARVEL		
Blood sample for Css of V and ODV, and mRNA			MARVEL		
Cocktail of drugs^a^ administration			MARVEL		
Blood sample for Phenotypic study			MARVEL		
Total amount of blood for the research	Visit 1 only: 12.5 mL of venous blood and 3 drops of capillary blood sample				

^a^fexofenadine, midazolam, dextromethorphan, omeprazole

*MADRS* (Montgomery and Asberg Depression Rating Scale), *HAM-D* (Hamilton Rating Scale for Depression), *QIDS* (Quick Inventory of Depressive Symptomatology), *MARS* (score Medication Adherence Report Scale), *PRISE-M* (Patient Rated Inventory of Side Effects), *FIBSER* (Frequency, Intensity, and Burden of Side Effects Ratings), *V* (Venlafaxine), *ODV* (O-demethyl-Venlafaxine)

#### The phenotype visit (V1)

The visit will take place between 7 and 21 days after Visit V0, in the morning.

The minimal delay between Visit V0 and Visit V1 will include the time to reach steady state of antidepressant drug since the increase in VLX dosage. Several criteria will be verified:

- Compliance with the prescribed medication regimen will be verified from the medication diary: an oversight of a single dose of VLX during the four days before V1 will exclude the patient.
- Minimum delay of 7 days between Visit V0 and Visit V1.
- No change in VLX dosage or co-medications (antipsychotics only) between inclusion and Visit V1.
- Negative urinary pregnancy test. The test will be performed for women of childbearing age in the morning before cocktail administration.
- No intake of usual drugs in the morning of the cocktail administration. Fasting state since almost 12 h. Last VLX intake 20–30 h before.

If the patient does not meet these criteria, they will be excluded from the study.

The nurse will then collect 4 venous blood samples before the administration of the substrate cocktail.

- Two samples of 5 ml for the concentration of VLX and ODV (heparinised capillary tubes)
- One sample of 7 mL for DNA collection (EDTA tube)
- One sample of 2.5 mL in PAXgen according to standardized procedure

In the morning after an overnight fast, the following cocktail of probe drugs are administered to the patients by a nurse: omeprazole 10 mg (capsule), dextromethorphane bromhydrate 10 mg (oral liquid formulation), midazolam 1 mg (injectable solution for oral administration), fexofenadine 120 mg (tablet). The pill and liquid formulations will be taken orally successively with a glass of water.

The blood samples will be collected as follow: Capillary blood samples at 2, 3 and 6 h after the cocktail administration (1 drops each hour) from a small finger prick will be collected using the Dried Blood Spot sampling method (Fig. 2), for the measurement of cocktail drug concentrations (drug parent and metabolites). To simplify the process of capillary blood collection, a device called HemaXis™ has been developed by DBS System SA (Switzerland) [37]. The HemaXis device integrates a patented microfluidic plate (WO/2013/144743) allowing accurate volume control and a conventional filter paper card for blood storage. Using this novel device, 10 μL-DBS samples can be easily generated from capillary blood drop without additional manipulation. Preliminary results show excellent performances in terms of precision of collected volume and ease of use.

In addition, alimentation during the preceding 7 days will be recorded by a standardized brief questionnaire. Patients will be questioned and monitored at each blood sampling: Diziness (yes/no); Headache (yes/no); Nausea, vomiting (yes/no), cardiac frequency (yes/no), systolic and diastolic tension. Breakfast will be possible 1 h after taking the cocktail drugs.

#### Follow-up visits (V2 and V3)

The visits V2 and V3 will take place between 25 and 40 days (4 weeks) and 50–70 days (8 weeks) after Visit V0, with a psychologist or a practitioner. They include assessment of efficacy, tolerance and observance. Once the V3 achieved, the study is finished for the patient who continues its usual follow-up.

Recruitment is expected to be completed within approximately 2 years.

### Assessment criteria

#### Primary assessment criteria

CYP2C19 activity: 5-hydroxyomeprazole/omeprazole at 2 h and AUC_2,3,6_ of the Metabolic ratio 2, 3 and 6 h after omeprazole oral administration.

CYP2D6 activity: dextrorphan/dextromethorphan ratio two hours after dextromethorphan oral administration.

CYP3A4 activity: 1-hydroxymidazolam/ midazolam ratio two hours after midazolam oral administration.

P-gp activity: Fexofenadine AUC_2,3,6_ based on fexofenadine concentrations at 2, 3 and 6 h after fexofenadine oral administration.

#### Antidepressant concentrations: VLX + ODV.

### Secondary assessment criteria

The MADRS is a ten-item diagnostic questionnaire, which psychiatrists use to measure the severity of depressive episodes in patients with mood disorders [38, 39]. It was designed in 1979 by British and Swedish researchers as an adjunct to the HAMD, which would be more sensitive to the changes brought on by antidepressants and other forms of treatment than the Hamilton Scale alone was. There is, however, a high degree of statistical correlation between scores on the two measures. Each item is coded 0 to 6 by the physician, the maximal score is 60; Depression is defined by a score ≥ 15. MADRS remission is defined by a score less than 10 and.

Patient responders to VLX are defined by a 50% decrease in MADRS score at 8 weeks of VLX treatment in comparison with MADRS score measured during patient selection.

The HAMD is a multiple item questionnaire used to provide an indication of depression, and as a guide to evaluate recovery (hedlund, hamiltton). The questionnaire is designed for adults and is used to rate the severity of their depression by probing mood, feelings of guilt, suicidal ideation, insomnia, agitation or retardation, anxiety, weight loss, and somatic symptoms. Initially considered the “Gold Standard” for rating depression in clinical research, this scale should not be used as a diagnostic instrument.

The original 1960 version contains 17 items to be rated (HRSD-17), but three other questions are not added to the total score and are used to provide additional clinical information. Although Hamilton’s original scale had 17 items, other versions were developed to include up to 29 items (HRSD-29). Each item on the questionnaire is scored on a 3 or 5-point scale, depending on the item, and the total score is compared to the corresponding descriptor. Remission of a MDD is defined as a HAM-D score of less than 10 (Williams 88).

The Medication Adherence Report Scale (MARS): The MARS scale is a ten-item self-report measure of medication adherence, initially developed for schizophrenia [40].

Brief scale for anxiety: The brief scale for anxiety of Tyrer is a subdivision of the comprehensive psychopathological scale [41]. It is a clinical interview rating scale designed to assess the psychology and somatic symptoms of anxiety; the interviewer rates the subject on each of 10 symptoms on a 7-point scale from 0 (no occurrence of the symptom) to 7 (incapacitation by lack of control of the symptom).

Criteria for rating medication trials for antidepressant failure: The Antidepressant Treatment History Form (ATHF) consists of scoring instructions and ratings for most antidepressants, augmentation and Electro convulsive therapy trials. It is being used increasingly to determine the adequacy of antidepressant trials.

Others: Most complaints listed as adverse reactions in people with depression are more common when they were medication-free rather than during their treatment with antidepressants [42]. The Frequency, Intensity, and Burden of Side Effects Rating (FIBSER) Scale, was developed to document these three domains of side effects in patients treated in the Sequenced Treatment Alternatives to Relieve Depression (STAR*D) project. The FIBSER is a reliable and valid self-report measure of side effects in a population receiving treatment for depression. Although it does not measure the impact of specific side effects, it does measure three domains of impact: frequency, intensity, and burden of the side effects. Its brevity makes it a useful tool for routine clinical practice.

Side effects are evaluated with the Patient Rated Inventory of Side Effects (PRISE-M) [43]. It is a 31-item checklist of side effects rated for the last 7 days, classified by symptom domains i.e. gastrointestinal, cardiac, skin, nervous system, eyes/ears, genital/urinary, sleep, sexual functioning, and other. Each domain has multiple symptoms that can be endorsed. For each domain the patient rates whether or not the symptoms are absent (0) tolerable [1] or distressing [2]. A total score defines a global side effects level, which takes into account the frequency and severity of each side effect. The frequency (% patients with the side effect tolerable or distressing) and severity of each side effect or of each domain can also be calculated. Patients with side effects are defined by a PRISE-M score > 10.

Fagerström Test measures nicotine dependence.

### Phenotypic analysis

#### From DBS to determination of phenotype

DBS devices will be frozen at −20 °C pending transport and analysis. The enzymatic activities will be assessed by specific metabolite/probe concentration ratios (metabolic ratios-MR) from a sample taken 2 h after cocktail administration for CYP2C19, CYP3A4, CYP2D6; and by the determination of the limited area under the curve (AUC) from the sample taken 2–3 and 6 h after cocktail administration for CYP2C19 and P-gp.

The cocktail substrates and their CYP-specific metabolites will be quantified in DBS using a single reverse- phase high-performance liquid chromatography–tandem mass spectrometry method operating in dual electrospray ionization mode, as previously described [44, 45]. The substances of interest will be extracted from DBS samples using methanol, whereas protein precipitation using acetonitrile will be used for plasma extraction. This method has been fully validated according to international criteria. The phenotype will be determined according to the results of the MR, and based on the results of previous studies [45].

#### Drug concentration

Blood samples will be centrifuged and serum will be collected in glass tubes. They will be frozen at −20 °C until transport and analysis. Plasma concentration will be quantified using Liquid chromatography coupled to UV visible diode array detector.

#### DNA collection and circulating mRNA

For DNA analysis, blood samples will be conserved in a 7 mL EDTA tube. For RNA analysis, blood samples will be conserved in PAXGEN tubes at ambient temperature for 2-72 h in a vertical position; they will then be frozen at −20 °C. Specific Genetic analyses will be decided upon at the end of the study.

### Statistics

Statistical analysis will be performed when the sample size has been reached, and all the end point measures available.

Remission rates with citalopram as the first step in STAR*D study were 28 to 33%, and response rates averaged 47% [46] after 14 weeks of treatment. After unsuccessful treatment with an SSRI, 28% of patients had a remission of symptoms after switching to VLX after 14 weeks of treatment. Schweitzer et al. observed, in patients suffering with moderate depression (MADRS = 32.8 at entry) that 69% were responders to VLX after 8 weeks of treatment and 36.7% were in remission. Hence, the proportion of remitters and responders vary according to the study; the prevalence of responders is higher than remitters and the time to assess these criteria also vary according to these studies. Schweitzeir et al. showed that in patients who were responders to VLX at 8 weeks, the response was maintained and even improved up to 10 months after [47]. Moreover, it is recognized that the antidepressant should be administered for 4 to 6 weeks before non-response can be assumed [48].

Given these data we estimate that response rates to VLX at 8 weeks will be 40%. We hypothesize that the prevalence of patients with a CYP2C19 UM profile is twice as high in non-responders in comparison with responders, who have a CYP2C19 metabolic profile comparable to that of Caucasians (20%). To demonstrate that the prevalence is two-fold that observed in non-responders, with a type I error at 0.05 and a statistical power of 80%, the sample size is tabulated below according to the prevalence of response (Table 2).

**Table 2:** Computation of sample size

Expected prevalence of responders	Number of responders	Number of non responders	Total sample size
50%	82	82	164
33%	63	125	188
28%	59	146	205

In anticipation of potential large disproportions between responders and non-responders (which will only be defined after study inclusion) we decided to include 205 patients. This will allow controlling for type I and typing II error rates in the comparison of the prevalence of CYP2C19 UMs among these groups.

In addition the sample size will allow to study sufficient numbers of CYP2D6 PMs, IMs, and UMs to determine the effects of CYP2D6 variations on VLX and ODV plasma levels and their efficacy or risk of adverse events.

The type I error rate will be fixed at 0.05. All tests will be two-sided and compared thus to 0.05.

Multiple imputation, which is a popular approach for handling the pervasive problem of missing data in biostatistics, will be used [49]. It is usually performed under a missing at random assumption [50]. Multiple imputations by chained equation are to our knowledge the most flexible approach to handle complex patterns of missing data (including categorical data, quantitative data, and survival data).

Primary analyses will be performed on an intent-to-treat basis. Secondary exploratory analyses will consider the population of compliers, that is, those who completed the treatment according to the scheduled protocol.

### Ethics

Ethical approval was obtained by an Independent Ethics Committee (CPP Ile de France I, Paris) and by the Agence Française de Sécurité Sanitaire des Produits de Santé (ANSM, French Health Products Safety Agency). The study was registered at the ClinicalTrials.gov website (NCT02590185).

## Discussion

In everyday practice, it remains difficult to accurately predict which patients will respond to which antidepressant and at which dose. Most of the research on the effect of metabolism and transport variability on antidepressants PK and PD was conducted either in healthy subjects or in patients using a genetic approach [8]; these studies often confirmed a relationship for a given drug/DME interaction but they did not investigate the cumulative effect of multiple metabolic pathways. Several studies concentrated on exploring the PK variability of tricyclic antidepressant, which currently are prescribed less frequently relative to other antidepressant classes.

The implementation of an assay able to predict the dose most likely to achieve maximal therapeutic benefit with minimal/tolerable adverse effects for a given patient may reduce the socioeconomic burden associated with suboptimal treatment. The Geneva Cocktail Phenotypic approach presents several advantages including the *“*in vivo*”* measure of different cytochromes and transporter P-gp activities, their simultaneous determination in a single test, avoiding the influence of variability over time on phenotyping results, the administration of low dose substrates, a limited sampling strategy with an analytical method developed on DBS analysis. By showing an association between drug metabolism and VLX concentrations, efficacy and tolerance, there is a hope that testing drug metabolism pathways with a phenotypical approach would help physicians in selecting and dosing antidepressants.

We recognize that therapeutic drug monitoring (TDM) may also help to find the right dosage for an individual patient, especially during start of therapy. But prior to and in addition to TDM, information about the activity of several metabolic pathways could further improve this dose finding and the choice of a given antidepressant.

A possible limitation of this study is that phenotyping is performed after the antidepressant has already been started for several days; antidepressants themselves are known to modify drug metabolism (ref), It would be ideal to evaluate the information before treatment has started. However this does not reflect clinical reality. Most patients still receive an antidepressant, with its respective influence on drug metabolism, when the psychiatrist takes the decision to switch. Waiting for the complete extinction of an inhibitory effect before performing a phenotypic determination of drug metabolism and before starting pharmacotherapy could delay pharmacotherapy and is, therefore, unwanted. Obviously a genotypic approach to defining drug metabolism is a very interesting method for exploring drug metabolism variability but has its own limits as detailed in the introduction. TDM of antidepressant, phenotyping and genotyping are complementary, and their combined use could contribute to improve the understanding of the determinants of the response to antidepressants.

Finally, the MARVEL study will provide an important contribution to increasing the knowledge of VLX variability and in optimizing the use of methods of personalized therapy in psychiatric settings.

## Abbreviations

5HTR2A: Serotonin 2A receptor gene
ANSM: Agence Française de Sécurité Sanitaire des Produits de Santé
ATHF: Antidepressant Treatment History Form
AUC: Area under the curve
CPP: Comité de protection des personnes
CYP: Cytochrome P450
DBS: Dried Blood Spot
DME: Drug-metabolizing enzymes
EM: Extensive metabolizer
FIBSER: Frequency, Intensity, and Burden of Side Effects Rating scale
HAM-D: Hamilton Rating Scale for Depression
IM: Intermediate metabolizer
MADRS: Montgomery and Asberg Depression Rating Scale
MARS: Medication Adherence Report Scale
MDD: Major depressive disorder
MR: Metabolic ratio
NDV: N-desmethyl-VLX
ODV: O-demethyl-venlafaxine
PD: Pharmacodynamic
P-gp: P-glycoprotein
PK: Pharmacokinetic
PM: Poor or slow metabolizer
PRISE-M: Patient Rated Inventory of Side Effects
SLC6A4: Serotonin transporter gene and the
STAR*D: Sequenced Treatment Alternatives to Relieve Depression project
TDM: Therapeutic drug monitoring
UM: Ultrarapid metabolizer
VLX: Venlafaxine

## References

[1] Global Burden of Disease Study C (2015). Global, regional, and national incidence, prevalence, and years lived with disability for 301 acute and chronic diseases and injuries in 188 countries, 1990-2013: a systematic analysis for the global burden of disease study 2013. Lancet.

[2] Sobocki P, Jonsson B, Angst J, Rehnberg C (2006). Cost of depression in Europe. J Ment Health Policy Econ.

[3] Gustavsson A, Svensson M, Jacobi F (2011). Cost of disorders of the brain in Europe 2010. Eur Neuropsychopharmacol.

[4] Souery D, Oswald P, Massat I (2007). Clinical factors associated with treatment resistance in major depressive disorder: results from a European multicenter study. J Clin Psychiatry.

[5] Souery D, Amsterdam J, de Montigny C (1999). Treatment resistant depression: methodological overview and operational criteria. Eur Neuropsychopharmacol.

[6] Kocsis JH. New strategies for treating chronic depression. J Clin Psychiatry 2000 61 Suppl 11):42–5. 10926054

[7] Crown WH, Finkelstein S, Berndt ER (2002). The impact of treatment-resistant depression on health care utilization and costs. J Clin Psychiatry.

[8] Lloret-Linares C, Bellivier F, Haffen E (2015). Markers of individual drug metabolism: towards the development of a personalized antidepressant prescription. Curr Drug Metab.

[9] Hall-Flavin DK, Winner JG, Allen JD, et al. Using a pharmacogenomic algorithm to guide the treatment of depression. Transl Psychiatry. 20122:e172. 10.1038/tp.2012.99 PMC3565829 23047243

[10] Altar CA, Carhart J, Allen JD, Hall-Flavin D, Winner J, Dechairo B (2015). Clinical utility of combinatorial pharmacogenomics-guided antidepressant therapy: evidence from three clinical studies. Mol Neuropsychiatry.

[11] Winner J, Allen JD, Altar CA, Spahic-Mihajlovic A. Psychiatric pharmacogenomics predicts health resource utilization of outpatients with anxiety and depression. Transl Psychiatry. 20133:e242. 10.1038/tp.2013.2 PMC3625917 23511609

[12] Winner JG, Carhart JM, Altar CA (2015). Combinatorial pharmacogenomic guidance for psychiatric medications reduces overall pharmacy costs in a 1 year prospective evaluation. Curr Med Res Opin.

[13] McGraw J, Waller D (2012). Cytochrome P450 variations in different ethnic populations. Expert Opin Drug Metab Toxicol.

[14] Zhou H, Tong Z, McLeod JF (2004). "Cocktail" approaches and strategies in drug development: valuable tool or flawed science?. J Clin Pharmacol.

[15] Fuhr U, Jetter A, Kirchheiner J (2007). Appropriate phenotyping procedures for drug metabolizing enzymes and transporters in humans and their simultaneous use in the "cocktail" approach. Clin Pharmacol Ther.

[16] Floyd MD, Gervasini G, Masica AL (2003). Genotype-phenotype associations for common CYP3A4 and CYP3A5 variants in the basal and induced metabolism of midazolam in European- and African-American men and women. Pharmacogenetics.

[17] Rebsamen MC, Desmeules J, Daali Y (2009). The AmpliChip CYP450 test: cytochrome P450 2D6 genotype assessment and phenotype prediction. Pharmacogenomics J.

[18] Heller T, Kirchheiner J, Armstrong VW (2006). AmpliChip CYP450 GeneChip: a new gene chip that allows rapid and accurate CYP2D6 genotyping. Ther Drug Monit.

[19] de Leon J, Armstrong SC, Cozza KL (2006). Clinical guidelines for psychiatrists for the use of pharmacogenetic testing for CYP450 2D6 and CYP450 2C19. Psychosomatics.

[20] Miller DB, O'Callaghan JP. Personalized medicine in major depressive disorder -- opportunities and pitfalls. Metabolism: clinical and experimental 2013 62 Suppl 1):S34–9. 10.1016/j.metabol.2012.08.021 PMC4672728 23021040

[21] Samer CF, Lorenzini KI, Rollason V, Daali Y, Desmeules JA (2013). Applications of CYP450 testing in the clinical setting. Mol Diagn Ther.

[22] Steiner E, Bertilsson L, Sawe J, Bertling I, Sjoqvist F (1988). Polymorphic debrisoquin hydroxylation in 757 Swedish subjects. Clin Pharmacol Ther.

[23] Sachse C, Brockmoller J, Bauer S, Roots I (1997). Cytochrome P450 2D6 variants in a Caucasian population: allele frequencies and phenotypic consequences. Am J Hum Genet.

[24] Rush AJ, Trivedi MH, Wisniewski SR (2006). Bupropion-SR, sertraline, or venlafaxine-XR after failure of SSRIs for depression. N Engl J Med.

[25] Cipriani A, Furukawa TA, Salanti G (2009). Comparative efficacy and acceptability of 12 new-generation antidepressants: a multiple-treatments meta-analysis. Lancet.

[26] Wellington K, Perry CM (2001). Venlafaxine extended-release: a review of its use in the management of major depression. CNS drugs.

[27] Hiemke C, Baumann P, Bergemann N (2011). AGNP consensus guidelines for therapeutic drug monitoring in psychiatry: update 2011. Pharmacopsychiatry.

[28] Sangkuhl K, Stingl JC, Turpeinen M, Altman RB, Klein TE (2014). PharmGKB summary: venlafaxine pathway. Pharmacogenet Genomics.

[29] Fogelman SM, Schmider J, Venkatakrishnan K (1999). O- and N-demethylation of venlafaxine in vitro by human liver microsomes and by microsomes from cDNA-transfected cells: effect of metabolic inhibitors and SSRI antidepressants. Neuropsychopharmacology.

[30] Veefkind AH, Haffmans PM, Hoencamp E (2000). Venlafaxine serum levels and CYP2D6 genotype. Ther Drug Monit.

[31] Klamerus KJ, Maloney K, Rudolph RL, Sisenwine SF, Jusko WJ, Chiang ST (1992). Introduction of a composite parameter to the pharmacokinetics of venlafaxine and its active O-desmethyl metabolite. J Clin Pharmacol.

[32] Shams ME, Arneth B, Hiemke C (2006). CYP2D6 polymorphism and clinical effect of the antidepressant venlafaxine. J Clin Pharm Ther.

[33] Hermann M, Hendset M, Fosaas K, Hjerpset M, Refsum H (2008). Serum concentrations of venlafaxine and its metabolites O-desmethylvenlafaxine and N-desmethylvenlafaxine in heterozygous carriers of the CYP2D6*3, *4 or *5 allele. Eur J Clin Pharmacol.

[34] Ball SE, Ahern D, Scatina J, Kao J (1997). Venlafaxine: in vitro inhibition of CYP2D6 dependent imipramine and desipramine metabolism; comparative studies with selected SSRIs, and effects on human hepatic CYP3A4, CYP2C9 and CYP1A2. Br J Clin Pharmacol.

[35] Jornil J, Nielsen TS, Rosendal I (2013). A poor metabolizer of both CYP2C19 and CYP2D6 identified by mechanistic pharmacokinetic simulation in a fatal drug poisoning case involving venlafaxine. Forensic Sci Int.

[36] McAlpine DE, Biernacka JM, Mrazek DA (2011). Effect of cytochrome P450 enzyme polymorphisms on pharmacokinetics of venlafaxine. Ther Drug Monit.

[37] Leuthold LA, Heudi O, Deglon J (2015). New microfluidic-based sampling procedure for overcoming the hematocrit problem associated with dried blood spot analysis. Anal Chem.

[38] Williams JB, Kobak KA (2008). Development and reliability of a structured interview guide for the Montgomery Asberg depression rating scale (SIGMA). Br J Psychiatry.

[39] Hawley CJ, Gale TM, Sivakumaran T (2002). Hertfordshire neuroscience research g. Defining remission by cut off score on the MADRS: selecting the optimal value. J Affect Disord.

[40] Thompson K, Kulkarni J, Sergejew AA (2000). Reliability and validity of a new medication adherence rating scale (MARS) for the psychoses. Schizophr Res.

[41] Lesur A, Bonnet D, Vicaut E, Lemperiere T (1989). Tyrer's brief scale for anxiety used with outpatients. First validation in the French language. Encéphale.

[42] Uher R, Farmer A, Henigsberg N (2009). Adverse reactions to antidepressants. Br J Psychiatry.

[43] Bryan C, Songer T, Brooks MM (2010). The impact of diabetes on depression treatment outcomes. Gen Hosp Psychiatry.

[44] Bosilkovska M, Deglon J, Samer C (2014). Simultaneous LC-MS/MS quantification of P-glycoprotein and cytochrome P450 probe substrates and their metabolites in DBS and plasma. Bioanalysis.

[45] Bosilkovska M, Samer CF, Deglon J, et al. Geneva cocktail for cytochrome P450 and P-glycoprotein activity assessment using dried blood spots. Clinical pharmacology and therapeutics*.* 2014. 10.1038/clpt.2014.83 PMC4151019 24722393

[46] Trivedi MH, Rush AJ, Wisniewski SR (2006). Evaluation of outcomes with citalopram for depression using measurement-based care in STAR*D: implications for clinical practice. Am J Psychiatry.

[47] Schweitzer I, Burrows G, Tuckwell V (2001). Sustained response to open-label venlafaxine in drug-resistant major depression. J Clin Psychopharmacol.

[48] Anderson IM, Ferrier IN, Baldwin RC (2008). Evidence-based guidelines for treating depressive disorders with antidepressants: a revision of the 2000 British Association for Psychopharmacology guidelines. J Psychopharmacol.

[49] Rubin. Multiple imputation for nonresponse in surveys. Wiley online library*.* 1987.

[50] Little R. Statistical analysis with missing data, 2nd edition. Wiley 2002.

